# Impact of 2021 European Academy of Neurology/Peripheral Nerve Society diagnostic criteria on diagnosis and therapy of chronic inflammatory demyelinating polyradiculoneuropathy variants

**DOI:** 10.1111/ene.16190

**Published:** 2024-01-02

**Authors:** Alberto De Lorenzo, Giuseppe Liberatore, Pietro Emiliano Doneddu, Fiore Manganelli, Dario Cocito, Chiara Briani, Raffaella Fazio, Anna Mazzeo, Angelo Schenone, Vincenzo Di Stefano, Giuseppe Cosentino, Girolama Alessandra Marfia, Luana Benedetti, Marinella Carpo, Massimiliano Filosto, Giovanni Antonini, Angelo Maurizio Clerici, Marco Luigetti, Sabrina Matà, Tiziana Rosso, Marta Lucchetta, Gabriele Siciliano, Giuseppe Lauria Pinter, Guido Cavaletti, Maurizio Inghilleri, Teresa Cantisani, Francesca Notturno, Dario Ricciardi, Francesco Habetswallner, Emanuele Spina, Erdita Peci, Alessandro Salvalaggio, Yuri Falzone, Camilla Strano, Luca Gentile, Elisa Vegezzi, Giorgia Mataluni, Stefano Cotti Piccinelli, Luca Leonardi, Angela Romano, Eduardo Nobile‐Orazio, Italian CIDP Database Study Group, Pietro Emiliano Doneddu, Alberto De Lorenzo, Giuseppe Liberatore, Eduardo Nobile ‐Orazio, Dario Cocito, Fiore Manganelli, Emanuele Spina, Enrica Pisano, Lucio Santoro, Daniele Velardo, Camilla Strano, Raffaella Fazio, Marta Ruiz, Mario Cacciavillani, Francesca Castellani, Chiara Briani, Filomena Caria, Massimiliano Filosto, Elisa Bianchi, Ettore Beghi, Elena Pinuccia Verrengia, Stefano Jann, Antonio Toscano, Luca Gentile, Massimo Russo, Anna Mazzeo, Luca Leonardi, Giovanni Antonini, Giuseppe Cosentino, Ilaria Callegari, Andrea Cortese, Giorgia Mataluni, Girolama Alessandra Marfia, Angelo Maurizio Clerici, Federica Scrascia, Marinella Carpo, Angelo Schenone, Luana Benedetti, Corrado Cabona, Alessandro Beronio, Erika Schirinzi, Gabriele Siciliano, Marco Luigetti, Patrizia Dacci, Giuseppe Lauria, Tiziana Rosso, Claudia Balducci, Guido Cavaletti, Mario Sabatelli, Erdita Peci, Stefano Cotti Piccinelli

**Affiliations:** ^1^ Neuromuscular and Neuroimmunology Unit IRCCS Humanitas Research Hospital Milan Italy; ^2^ Department of Biomedical Sciences Humanitas University Milan Italy; ^3^ Department of Neuroscience, Reproductive Sciences, and Odontostomatology University of Naples "Federico II" Naples Italy; ^4^ Department of Neuroscience University of Turin Turin Italy; ^5^ Neurology Unit, Department of Neuroscience University of Padua Padua Italy; ^6^ Division of Neuroscience, Department of Neurology, Institute of Experimental Neurology San Raffaele Scientific Institute Milan Italy; ^7^ Department of Clinical and Experimental Medicine, Unit of Neurology University of Messina Messina Italy; ^8^ Neurology Clinic IRCCS Ospedale Policlinico San Martino Genova Genoa Italy; ^9^ Department of Neuroscience, Rehabilitation, Ophthalmology, Genetics, Maternal and Child Health University of Genoa and IRCCS AOU San Martino‐IST Genoa Italy; ^10^ Department of Biomedicine, Neuroscience, and Advanced Diagnostics University of Palermo Palermo Italy; ^11^ Department of Brain and Behavioral Sciences University of Pavia Pavia Italy; ^12^ IRCCS Mondino Foundation Pavia Italy; ^13^ Dysimmune Neuropathies Unit, Department of Systems Medicine Tor Vergata University of Rome Rome Italy; ^14^ Department of Neurology ASST Bergamo Ovest‐Ospedale Treviglio Treviglio Italy; ^15^ Center for Neuromuscular Diseases and Neuropathies, Unit of Neurology, ASST "Spedali Civili" University of Brescia Brescia Italy; ^16^ Unit of Neuromuscular Diseases, Department of Neurology Mental Health and Sensory Organs, Faculty of Medicine and Psychology "Sapienza" University of Rome, Sant'Andrea Hospital Rome Italy; ^17^ Neurology Unit, Circolo and Macchi Foundation Hospital University of Insubria Varese Italy; ^18^ Neurology Department, Fondazione Policlinico Universitario Agostino Gemelli IRCCS Università Cattolica del Sacro Cuore Rome Italy; ^19^ Neurology Unit, Dipartimento Neuromuscoloscheletrico e Degli Organi di Senso University Hospital Careggi Florence Italy; ^20^ UOC di Neurologia, Ospedale San Bassano Vicenza Italy; ^21^ UOC Neurologia, Ospedale Santa Maria della Misericordia Rovigo Italy; ^22^ Neurology Unit, Department of Clinical and Experimental Medicine University of Pisa Pisa Italy; ^23^ Unit of Neuroalgology IRCCS Foundation "Carlo Besta" Neurological Institute Milan Italy; ^24^ Department of Medical Biotechnology and Translational Medicine Milan University Milan Italy; ^25^ School of Medicine and Surgery and Experimental Neurology Unit University of Milano‐Bicocca Monza Italy; ^26^ Neurodegenerative Diseases Unit, Department of Human Neuroscience Sapienza University, Policlinico Universitario Umberto I Rome Italy; ^27^ Servizio di Neurofisiopatologia Azienda Ospedaliera di Perugia Perugia Italy; ^28^ UOC Neurologia, Ospedale Santi Filippo e Nicola Avezzano Italy; ^29^ Division of Neurology and Neurophysiopathology, Department of Medical and Surgical Sciences University of Campania "Luigi Vanvitelli" Naples Italy; ^30^ Clinical Neurophysiology Unit Cardarelli Hospital Naples Italy

**Keywords:** chronic inflammatory demyelinating polyradiculoneuropathy, chronic inflammatory demyelinating polyradiculoneuropathy variants, diagnostic criteria, European Academy of Neurology, intravenous immunoglobulin, Lewis–Sumner syndrome, Peripheral Nerve Society, steroids

## Abstract

**Background and purpose:**

There are different criteria for the diagnosis of different variants of chronic inflammatory demyelinating polyradiculoneuropathy (CIDP). The 2021 European Academy of Neurology/Peripheral Nerve Society (EAN/PNS) guidelines provide specific clinical criteria for each CIDP variant even if their therapeutical impact has not been investigated.

**Methods:**

We applied the clinical criteria for CIDP variants of the 2021 EAN/PNS guidelines to 369 patients included in the Italian CIDP database who fulfilled the 2021 EAN/PNS electrodiagnostic criteria for CIDP.

**Results:**

According to the 2021 EAN/PNS clinical criteria, 245 patients achieved a clinical diagnosis of typical CIDP or CIDP variant (66%). We identified 106 patients with typical CIDP (29%), 62 distal CIDP (17%), 28 multifocal or focal CIDP (7%), four sensory CIDP (1%), 27 sensory‐predominant CIDP (7%), 10 motor CIDP (3%), and eight motor‐predominant CIDP (2%). Patients with multifocal, distal, and sensory CIDP had milder impairment and symptoms. Patients with multifocal CIDP had less frequently reduced conduction velocity and prolonged F‐wave latency and had lower levels of cerebrospinal fluid protein. Patients with distal CIDP more frequently had reduced distal compound muscle action potentials. Patients with motor CIDP did not improve after steroid therapy, whereas those with motor‐predominant CIDP did. None of the patients with sensory CIDP responded to steroids, whereas most of those with sensory‐predominant CIDP did.

**Conclusions:**

The 2021 EAN/PNS criteria for CIDP allow a better characterization of CIDP variants, permitting their distinction from typical CIDP and more appropriate treatment for patients.

## INTRODUCTION

Chronic inflammatory demyelinating polyradiculoneuropathy (CIDP) is a chronic inflammatory neuropathy with a broad spectrum of clinical heterogeneity [1, 2]. Clinical variants of CIDP, previously known as atypical CIDP, have been described and characterized in literature and include distal acquired demyelinating symmetric polyneuropathy (DADS), Lewis–Sumner syndrome (LSS), focal CIDP, and pure motor or pure sensory CIDP [3, 4, 5, 6, 7, 8, 9, 10, 11, 12]. It is still unclear whether these variants represent a different phenotypical presentation of the same disease, a step that precedes the progression to typical CIDP [13], or separate clinical entities with different response to therapy and, possibly, a different pathogenic mechanism. The last possibility is supported by pathological and electrophysiological differences between CIDP variants and typical CIDP [5, 14]. Moreover, specific cytokine patterns have been identified in LSS, which may reflect a distinct underlying pathogenesis [15, 16]. On the other hand, the discovery of antibodies against components of the node and paranode has shown that there are patients with different clinical forms of CIDP sharing the presence of the same antibody and patients with similar clinical characteristics that differ in their antibody status [17]. All this recent evidence has led some authors to suggest that the discovery of antibodies leads to a break with traditional clinical CIDP classification [18].

Recently, a second revision of the European Federation of Neurological Societies/Peripheral Nerve Society (EFNS/PNS) criteria has been published in 2021 and named the European Academy of Neurology and Peripheral Nerve Society (EAN/PNS) criteria. These criteria provided more specific clinical and electrophysiological criteria for each CIDP variant [19], named distal CIDP, multifocal and focal CIDP, sensory CIDP, sensory‐predominant CIDP, motor CIDP, and motor‐predominant CIDP. In addition, the 2021 EAN/PNS criteria excluded patients with anti‐nodal/paranodal antibodies from CIDP, including them under the term nodo‐paranodopathy [18]. To date, no studies have evaluated whether the 2021 EAN/PNS criteria permit a better clinical, electrophysiological, and therapeutic definition of the individual CIDP forms compared to previous criteria.

## METHODS

### Study design

We compared the clinical and electrophysiological characteristics and treatment response of the patients diagnosed with CIDP variants with those of the patients diagnosed with typical CIDP using the 2021 EAN/PNS clinical and electrophysiological criteria.

### Database and study population

We implemented a web‐based registry of Italian CIDP patients where data from patients with a diagnosis of typical CIDP or its variants were included. All data were included by the treating neurologist in a web‐based electronic database expressly prepared by CINECA, Bologna, Italy. The diagnosis of CIDP was reviewed by the coordinating center (P.E.D. and E.N.‐O.) in accordance with the treating neurologist, classified according to the 2010 EFNS/PNS diagnostic criteria, and subsequently reviewed according to the 2021 EAN/PNS criteria (A.D.L.).

We decided that a minimum of 1‐year duration of symptoms and signs specific to each CIDP form was necessary to establish a diagnosis of typical CIDP or its variants. This decision was made because even typical CIDP may initially present with purely sensory or motor symptoms, evolving over a few months to a typical sensorimotor form [13].

In this study, we employed the same methodology as reported in a previous study [13]. At enrollment, all eligible patients underwent a detailed clinical history that included information about the time of onset, distribution, and progression of signs and symptoms including weakness, sensory symptoms, ataxia, pain, cramps, tremor, fatigue, and cranial nerve impairment. This information was integrated with data recorded in the patients' medical records.

The treating neurologist defined the course of the disease as monophasic, progressive, or relapsing. A relapsing course was defined as a clinical worsening after an initial improvement that was not related to treatment suspension or dose reduction [20]. However, some patients with a delayed worsening (>3 months) after treatment suspension or reduction might also have been included in this group [20]. An acute onset of CIDP was also reported and defined as a neuropathy that was initially diagnosed as Guillain–Barré syndrome (GBS) but that continued to progress or relapse >2 months after disease onset.

The clinical evaluation at registry enrollment included assessment of muscle strength using the Medical Research Council (MRC) sum score on 12 muscles (range = 0–60). Neurological disability was evaluated at enrollment using the Inflammatory‐Rash Overall Built Disability Scale (raw score, range = 1–48) and the Inflammatory Neuropathy Cause and Treatment (INCAT) disability scale (range = 0–10). Quality of life (QoL) was assessed using the EuroQol‐5D‐3L scale, a standardized questionnaire assessing responses to five dimensions (mobility, self‐care, usual activities, pain or discomfort, and anxiety or depression), each with a score from 1 (best) to 3 (worst). No barometer scale was used for overall estimation of QoL.

The results of diagnostic nerve conduction studies (NCS) performed during the course of the disease as part of routine clinical care were also included. The NCS data of each patient included in the database were reviewed by the coordinating center and, in the case of missing or nondiagnostic data, a complete NCS examination was requested. Motor nerve conduction studies were asked to be performed bilaterally in the median, ulnar, common peroneal, and tibial nerves and to include distal and proximal (up to the elbow in most patients) compound muscle action potential (CMAP) amplitude (onset to peak) and duration, motor conduction velocities, distal and proximal motor latencies, and F‐wave latency. Sensory conduction studies were asked to be performed bilaterally in the median, ulnar, and sural nerves and to include sensory action potential amplitude, distal latency, and conduction velocity. All nerve conductions were performed at a temperature of at least 33°C at the palm and 30°C at the external malleolus. Results were analyzed according to each laboratory's range of normal values, and demyelinating parameters were defined according to the 2021 EAN/PNS electrodiagnostic criteria. To evaluate temporal dispersion, NCS waveforms of the CIDP patients were reviewed and measurements were redone following the indications of the 2021 EAN/PNS criteria [19]. Patients for whom nerve conduction study waveforms were not available for revision were excluded from the analysis of temporal dispersion.

Results of previously performed examinations, including cerebrospinal fluid (CSF) analysis and sural nerve biopsy, were reported when available. As to CSF protein counts, we considered as upper reference limit 50 mg/dL for patients aged ≤50 years and 60 mg/dL for those aged >50 years [21].

Response to treatment was defined as a subjective improvement that was objectively confirmed by an increase of at least 2 points on the MRC sum score (range = 0–60) or at least 1 point on the INCAT score (range = 0–10) [22, 23]. The response to treatment was evaluated prospectively by the treating neurologist and reported in the database.

Informed consent was obtained from all participants at enrollment, and the ethical committee of each participating center approved the study. The data that support the findings of this study are available from the corresponding author upon request.

### Statistical analysis

Categorical variables were described using frequency and percentage and analyzed with the chi‐squared or Fisher exact test. Continuous variables were described using mean and SD, assessed for normality with the Shapiro–Wilk test and analyzed with the *t*‐test (for normally distributed variables) or Wilcoxon–Mann–Whitney test (for nonparametrically distributed variables). Significance was set at an α‐level of 0.05, and no multiple testing correction was applied. The statistical analyses were performed using SPSS Statistics for Windows, version 28.0 (IBM, Armonk, NY, USA).

## RESULTS

### Patient selection and diagnostic criteria

**FIGURE 1:**
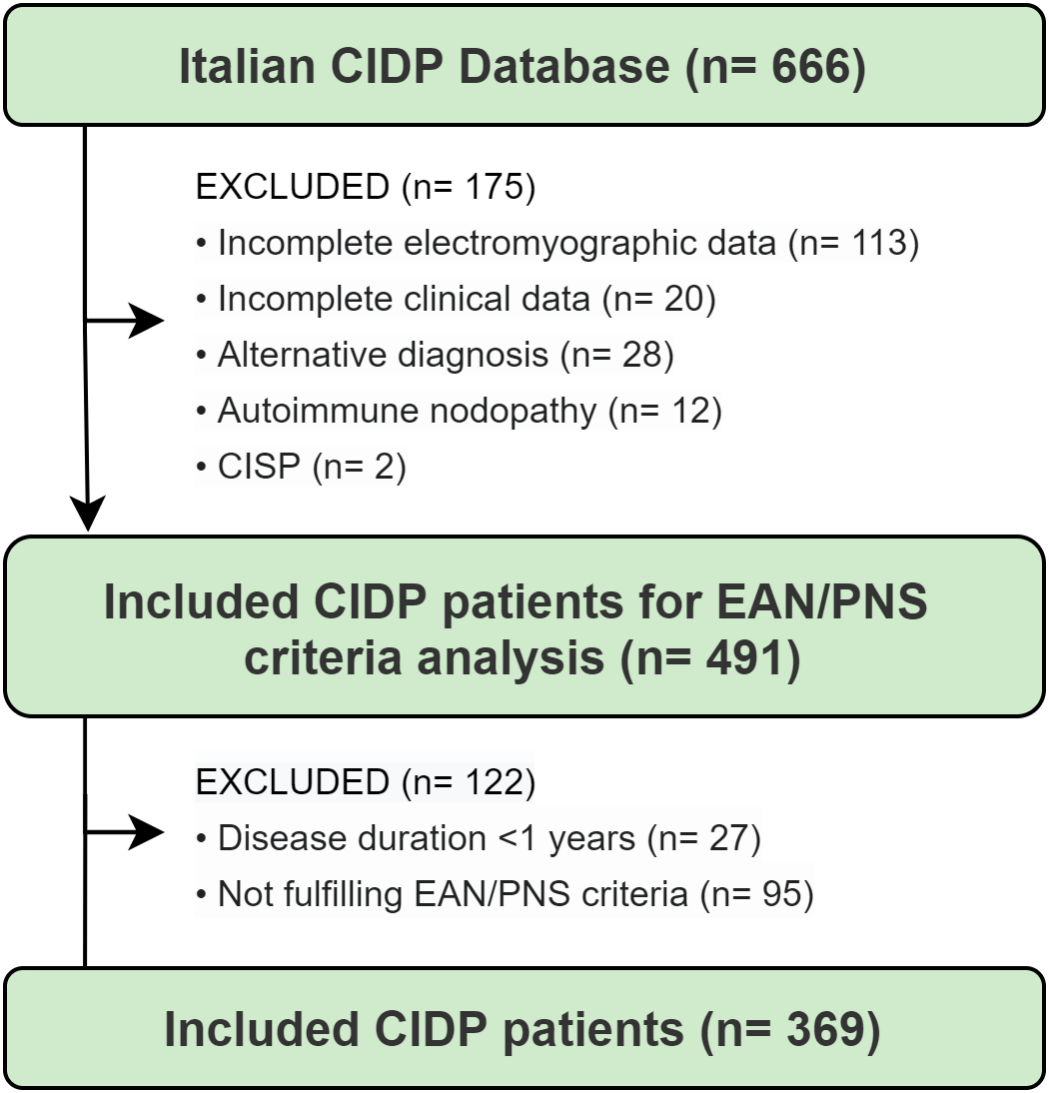
Case selection flowchart. CIDP, chronic inflammatory demyelinating polyradiculoneuropathy; CISP, chronic immune sensory polyradiculopathy; EAN/PNS, European Academy of Neurology/Peripheral Nerve Society.

The case selection process is summarized in Figure 1. By February 2023, 666 patients were enrolled in our database. Of the initial population, 133 patients were excluded for incomplete clinical or electrophysiological data, and 28 for having an alternative diagnosis (24 anti‐myelin‐associated glycoprotein antibody neuropathy and four amyloid neuropathy). In accordance with the 2021 EAN/PNS guidelines, we also excluded 12 patients with autoimmune nodopathy and two patients with chronic immune sensory polyradiculopathy. After excluding patients with a disease duration of <1 year (*n* = 27) and patients not fulfilling the 2021 EAN/PNS electrodiagnostic criteria for possible CIDP or CIDP (*n* = 162), a final study population of 369 patients (329 CIDP, 40 possible CIDP) were included in the analysis.

The study population included 234 males and 135 females, aged 12–92 years (mean = 58, median = 60 years), with a mean disease duration of 8 years (range = 1–52 years, median = 6). Mean time from symptom onset to NCS was 5.3 years (median = 1.9, SD = 7.72).

**FIGURE 2:**
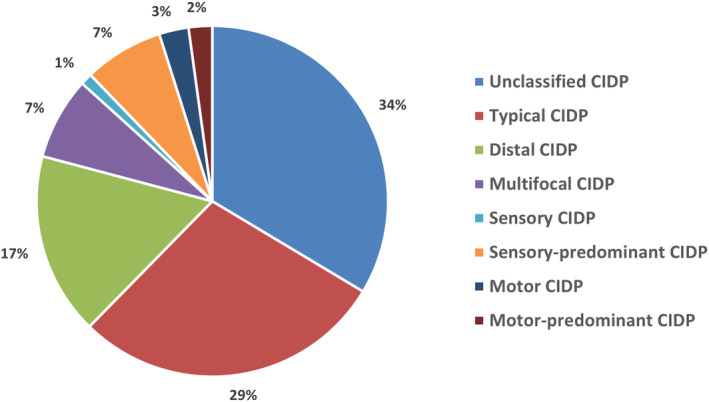
Clinical diagnosis of chronic inflammatory demyelinating polyradiculoneuropathy (CIDP) according to the 2021 European Academy of Neurology/Peripheral Nerve Society (EAN/PNS) clinical criteria for CIDP in 369 patients fulfilling the 2021 EAN/PNS electrodiagnostic criteria for possible CIDP.

Figure 2 shows the frequency of typical CIDP and CIDP variants at study entry. Notably, 124 (34%) patients fulfilled the electrodiagnostic criteria but did not strictly fulfill the clinical criteria for either typical CIDP or its variants and were included under the definition of “unclassified CIDP.” Features of this population have been reported in a separate study [24]. Of the remaining 245 patients, according to the 2021 EAN/PNS clinical criteria, 106 patients (29% of total 369 patients) had a diagnosis of typical CIDP, 62 (17%) had distal CIDP, 28 (7%) had multifocal or focal CIDP, four (1%) had sensory CIDP, 27 (7%) had sensory‐predominant CIDP, 10 (3%) had motor CIDP, and eight (2%) had motor‐predominant CIDP.

### Comparison of the clinical and electrophysiological characteristics and treatment response of CIDP variants and typical CIDP

The clinical and electrophysiological features of CIDP patients diagnosed according to the 2021 EAN/PNS criteria are summarized in Table 1. Each CIDP variant was compared to the typical CIDP population.

**TABLE 1:** Features of clinical variants defined according to EAN/PNS 2021 criteria.

Features	Typical CIDP, *n* = 106	Distal CIDP, *n* = 62	Multifocal and focal CIDP, *n* = 28	Sensory CIDP, *n* = 4	Sensory‐predominant CIDP, *n* = 27	Motor CIDP, *n* = 10	Motor‐predominant CIDP, *n* = 8	*p*
Demographics								
Gender male, *n* (%)	72/106 (68%)	37/62 (60%)	24/28 (86%)	2/2 (50%)	22/27 (82%)	5/10 (50%)	5/8 (63%)	
Age at onset, years, median (IQR)	50 (28)	58 (23)	54 (25)	55 (29)	61 (13)	59 (22)	30 (37)	0.027 D‐T, <0.001 SP‐T, 0.023 MP‐T
Disease duration at enrollment, years, median (IQR)	7 (10)	6 (8)	5 (6)	7 (4)	3 (4)	6 (15)	4 (10)	<0.001 MF‐T, 0.018 SP‐T
Disease course, *n* (%)								
Relapsing	49/106 (46%)	35/62 (57%)	11/28 (39%)	0/4 (0%)	10/27 (49%)	6/10 (60%)	5/8 (63%)	
Acute onset	11/106 (10%)	3/62 (5%)	0/28 (0%)	0/4 (0%)	0/24 (0%)	0/10 (0%)	3/8 38 (%)	
Symptoms, *n* (%)								
Pain	39/106 (37%)	22/62 (36%)	4/28 (14%)	1/4 (25%)	9/27 (33%)	6/10 (60%)	1/8 (13%)	0.023 MF‐T
Fatigue	68/106 (64%)	34/62 (55%)	6/28 (21%)	2/4 (50%)	8/27 (30%)	8/10 (80%)	6/8 (75%)	<0.001 MF‐T, 0.002 SP‐T
Cranial nerve involvement	30/106 (28%)	0/62 (0%)	6/28 (21%)	1/4 (25%)	0/27 (0%)	2/10 (20%)	1/8 (13%)	<0.001 D‐T, <0.001 SP‐T
Ataxia	44/106 (42%)	18/62 (29%)	4/28 (14%)	1/4 (25%)	6/27 (22%)	0/10 (0%)	0/8 (0%)	0.008 MF‐T, 0.013 M‐T, 0.022 MP‐T
Tremor	17/106 (16%)	8/62 (13%)	3/28 (11%)	1/4 (25%)	0/27 (0%)	1/10 (10%)	1/8 (13%)	0.026 SP‐T
Clinical scores at enrollment (±SD)								
INCAT	3.5 (±2.3)	2.2 (±1.7)	2.1 (±1.3)	0.5 (±1)	1.3 (±1)	3.3 (±2.8)	2.8 (±2.5)	<0.001 D‐T, <0.001 MF‐T, <0.001 S‐T, <0.001 SP‐T
MRC	52 (±10)	55 (±7)	56 (±4)	60 (±0)	60 (±0)	55 (±3)	53 (±8)	0.003 D‐T, <0.001 MF‐T, <0.001 S‐T, <0.001 SP‐T
RODS	32 (±12)	35 (±9)	37 (±10)	35 (±11)	41 (±6)	36 (±9)	34 (±11)	0.029 D‐T, <0.001 SP‐T
EuroQol‐5D‐3L scale	8.3 (±2.0)	7.6 (±1.5)	8.0 (±1.8)	9.2 (±1.0)	7.1 (±1.4)	7.5 (±1.9)	7.8 (±2.0)	0.032 D‐T, 0.019 SP‐T
Electromyographic features								
Tested motor nerves, *n*, median (IQR)	6 (2)	6 (2)	6 (3)	7 (4)	6 (2)	7 (2)	6 (4)	
Distal latency prolongation, *n* (%)	32/106 (30%)	24/62 (39%)	6/28 (21%)	0/4 (0%)	9/27 (33%)	1/10 (10%)	2/8 (25%)	
Reduced conduction velocity, *n* (%)	51/106 (48%)	35/62 (57%)	4/28 (14%)	0/4 (0%)	8/27 (30%)	2/10 (20%)	3/8 (38%)	0.001 MF‐T
Prolonged F‐wave latency, *n* (%)	19/80 (24%)	10/48 (21%)	1/23 (4%)	0/4 (0%)	8/24 (33%)	4/10 (40%)	3/7 (43%)	0.040 MF‐T
Absent F wave, *n* (%)	17/80 (21%)	5/48 (10%)	6/23 (26%)	0/4 (0%)	5/24 (21%)	2/10 (20%)	2/7 (29%)	
Conduction block, *n* (%)	39/100 (39%)	21/55 (38%)	5/25 (20%)	0/4 (0%)	5/25 (20%)	2/10 (20%)	3/7 (43%)	
Temporal dispersion, *n* (%)	29/60 (48%)	14/37 (38%)	5/16 (31%)	0/4 (0%)	5/18 (28%)	2/6 (33%)	2/3 (67%)	
Reduced CMAP amplitude, *n* (%)	55/106 (52%)	43/62 (69%)	10/28 (36%)	0/4 (0%)	16/25 (64%)	5/10 (50%)	6/8 (75%)	0.027 D‐T
Sural sparing pattern on sensory NCS, *n* (%)	20/75 (27%)	12/41 (29%)	8/20 (40%)	2/3 (66%)	9/22 (41%)	0/6 (0%)	2/8 (25%)	
Supportive criteria								
Increased CSF proteins/tested, *n* (%)	72/92 (78%)	37/46 (80%)	12/22 (55%)	2/3 (67%)	16/24 (67%)	4/6 (67%)	5/5 (100%)	0.023 MF‐T
Mean CSF proteins, mg/dL (±SD)	113 (±130)	103 (±71)	64 (±35)	63 (±28)	88 (±77)	201 (±335)	284 (±266)	0.003 MF‐T
Demyelination on nerve biopsy/tested, *n* (%)	4/6 (67%)	2/5 (40%)	0/1 (0%)	1/2 (50%)	ND	1/1 (100%)	ND	
Response to therapy								
Response IVIg/treated, *n* (%)	56/88 (64%)	36/48 (75%)	11/21 (52%)	2/4 (50%)	7/11 (64%)	6/10 (60%)	4/6 (67%)	
Response steroids/treated, *n* (%)	44/80 (55%)	20/34 (59%)	8/16 (50%)	0/2 (0%)	8/11 (73%)	0/3 (0%)	2/2 (100%)	
Overall response/treated, *n* (%)	76/100 (76%)	45/55 (82%)	19/26 (73%)	2/4 (50%)	14/17 (82%)	7/10 (70%)	6/7 (86%)	

Abbreviations: CIDP, chronic inflammatory demyelinating polyradiculoneuropathy; CMAP, compound muscle action potential; CSF, cerebrospinal fluid; D‐T, distal CIDP vs. typical CIDP; INCAT, Inflammatory Neuropathy Cause and Treatment; IQR, interquartile range; IVIg, intravenous immunoglobulin; MF‐T, multifocal CIDP vs. typical CIDP; MP‐T, motor‐predominant CIDP vs. typical CIDP; MRC, Medical Research Council; M‐T, motor CIDP vs. typical CIDP; NCS, nerve conduction studies; ND, not done; RODS, Rasch‐built Overall Disability Scale; SP‐T, sensory‐predominant CIDP vs. typical CIDP; S‐T, sensory CIDP vs. typical CIDP.

Distal CIDP patients were characterized by an older age at onset (54 vs. 48 years, *p* = 0.027), no cranial nerve involvement (0% vs. 28%, *p* < 0.001), less impairment and disability measured by the MRC sum score (55 vs. 52, *p* = 0.003), INCAT score (2.2 vs. 3.5, *p* < 0.001), and Rasch‐built Overall Disability Scale (RODS; 35 vs. 32, *p* = 0.029), better quality of life on the EuroQoL‐5D‐3L scale (7.6 vs. 8.3, *p* = 0.032), and more frequent distal CMAP amplitude reduction on NCS (69% vs. 52%, *p* = 0.027).

Patient with multifocal/focal CIDP had shorter disease duration at enrollment (5 vs. 10 years, *p* < 0.001), and less frequently reported pain (14% vs. 37%, *p* = 0.023), fatigue (21% vs. 64%, *p* < 0.001), and ataxia (14% vs. 42%, *p* = 0.008). They had less severe impairment and disability by the MRC sum score (mean = 56 vs. 52, *p* < 0.001) and INCAT score (mean = 2.1 vs. 3.5, *p* < 0.001), less frequently reduced motor conduction velocities (14% vs. 48%, *p* = 0.001) and prolonged F‐wave latency (4% vs. 24%, *p* = 0.040). They also had less frequently increased CSF proteins (55% vs. 78%, *p* = 0.023) and had lower CSF protein levels (64 vs. 113 mg/dL, *p* = 0.003).

Patients diagnosed with sensory CIDP had less severe impairment and disability by the MRC sum score (60 vs. 52, *p* < 0.001) and INCAT score (0.5 vs. 2.5, *p* < 0.001). Patients with sensory‐predominant CIDP had a shorter disease duration at enrollment (5 vs. 10 years, *p* = 0.018), were older at disease onset (mean = 58 vs. 48, *p* < 0.001), less frequently reported fatigue (30% vs. 64%, *p* = 0.002), and did not report cranial nerve involvement (0% vs. 28%, *p* < 0.001) or tremor (0% vs. 16%, *p* = 0.026). They had less severe impairment and disability by the MRC sum score (mean = 60 vs. 52, *p* < 0.001), INCAT score (mean = 1.3 vs. 3.5, *p* < 0.001), and RODS (mean = 41 vs. 32, *p* < 0.001), and reported better QoL on the EuroQoL‐5D‐3L scale (7.1 vs. 8.3, *p* = 0.019). Steroid therapy was administered to two sensory CIDP patients, without an evident clinical response, whereas eight of 11 patients (73%) with sensory‐predominant CIDP improved after steroid therapy.

Patients with motor and motor‐predominant CIDP did not report ataxia (0% vs. 42%, *p* = 0.013 and *p* = 0.022), and those with motor‐predominant CIDP had a younger age of disease onset (33 vs. 48 years, *p* = 0.023). None of three patients with pure motor CIDP treated with steroids improved after this therapy, whereas both treated patients with motor‐predominant CIDP improved after this therapy.

## DISCUSSION

The 2021 EAN/PNS clinical criteria provided clear and detailed definitions of each CIDP variant, representing a step forward compared to the 2010 EFNS/PNS criteria, where these variants were not clearly defined. The EAN/PNS criteria were also found to be more specific but less sensitive than the EFNS/PNS criteria [25, 26]. Among the changes made in the EAN/PNS criteria compared to the EFNS/PNS criteria, some were found to be disadvantageous, whereas others were effective in terms of diagnostic gain [25]. In a disease causing severe disability and for which therapies may be expensive, such as CIDP, both under‐ and overdiagnosis are inconvenient. We recently reported that a large proportion of patients fulfilling the 2021 EAN/PNS electrodiagnostic (and in some cases also the supportive) criteria for CIDP do not strictly meet the clinical criteria. These forms have been termed unclassified CIDP [24]. In our population, the combined frequency of CIDP variants defined according to the 2021 EAN/PNS guidelines (57%) was higher compared to that of typical CIDP (43%) and to what was previously reported using the 2010 EFNS/PNS criteria (18%) [13]. This difference mainly reflects the exclusion of patients with unclassified CIDP from the typical CIDP group, as the inclusion of these patients would have led to a proportion of patients diagnosed with CIDP variants of 38% versus 62% with typical CIDP. Approximately 90% of the patients with unclassified CIDP in our cohort had clinical presentation resembling typical CIDP but in which some segments of the four limbs (e.g., proximal areas of the upper limbs) were unaffected by weakness ("incomplete typical CIDP") [24].

Several recent lines of evidence suggest that typical CIDP and its variants may have differences in their pathogenesis. Electrophysiological, nerve imaging, and nerve biopsy studies have shown for instance that the distribution of lesions and the pattern of demyelination in the peripheral nervous system are different among the individual CIDP forms [5, 14]. Our study confirmed previous reports of milder symptomatology and lower levels of disability and impairment in patients with distal, multifocal, sensory, and sensory‐predominant CIDP [4, 5, 13, 14, 27]. Focal and sensory‐predominant CIDP presented a shorter disease duration at enrollment, which may, in part, be attributed to the phenotypic progression to typical CIDP commonly observed among CIDP variants following disease onset [13]. In addition, patients with multifocal CIDP had lower levels of CSF proteins, and less frequent reduced motor conduction velocity and prolonged F‐wave latencies. Even if motor conduction blocks are considered a common feature in LSS, their frequency was not significantly higher in our population. We also did not observe a different response to immune therapies in patients with multifocal CIDP compared to those with typical CIDP. Unclassified CIDP forms were reported to show a better treatment response compared to typical CIDP [24]. Their exclusion from the typical CIDP group may explain the lower than expected treatment response in the typical CIDP group, hence leading to the overestimation of treatment response of clinical variants [25]. When we repeated the analysis including patients with unclassified forms in the typical CIDP group, the response to therapy was higher than in patients with multifocal CIDP (75% vs. 52%, *p* = 0.029). These findings reinforce previous electrophysiological and pathological evidence indicating that in patients with LSS/multifocal CIDP, lesions are preferentially localized in the middle nerve trunk, suggesting that a different pathogenic mechanism may be involved in LSS/multifocal CIDP compared to typical CIDP [5, 14, 27]. The lower treatment responses observed in the CIDP variants may, however, also be attributed to their higher baseline MRC and INCAT scores compared to typical CIDP, which could limit the extent of observable improvement by these assessment metrics.

The results of previous studies on the response to therapy in DADS were quite controversial, with some studies showing a reduced response to therapy and to intravenous immunoglobulin (IVIg) compared to typical CIDP [5, 13], whereas others did not [9, 28]. There was also some heterogeneity in the reported distribution of electrophysiological and pathological abnormalities, with some studies showing similar features to typical CIDP and others to multifocal CIDP [5, 9, 28]. With the only exception of a more frequent presence of distal CMAP amplitude reduction, we did not find significant difference in electrophysiological findings and response to therapy in patients with distal CIDP and CIDP, similarly to what was initially reported in DADS [9]. Nevertheless, it is worth noting that the 2021 EAN/PNS criteria require the presence of both distal sensory and motor symptoms for the definition of distal CIDP, whereas in previous studies patients presenting with exclusively distal sensory symptoms were often classified as DADS. For this reason, it is not possible to conduct a conclusive comparison with previous literature on DADS in terms of clinical and neurophysiological features. Moreover, the recorded frequency of distal CIDP in this case series was higher than what was previously reported in the same population when different criteria were applied [13]; the main reason for this difference is that the criteria used for the definition of DADS in prior reports required mandatory lower limb onset and confinement for a minimum of 1 year, whereas the novel 2021 EAN/PNS criteria provide a less stringent clinical definition of distal involvement and do not exclude early upper limb involvement.

Motor CIDP patients displayed a lower age at onset and did not respond to steroid therapy. These findings align with previous reports on motor CIDP [10, 29] and the 2021 EAN/PNS criteria recommendation to use IVIg as first‐line therapy for motor CIDP. Conversely, motor‐predominant CIDP patients exhibited a positive response to steroid therapy. Although this observation was limited to a small number of patients, it provides further evidence that only motor CIDP, similarly to multifocal motor neuropathy (MMN) [30], is refractory to steroid therapy, whereas motor‐predominant CIDP is not, supporting the role of sensory NCS in differentiating the two conditions. This finding led us to consider that motor CIDP might be a symmetric variant of MMN more than a variant of CIDP and that probably stays at MMN as multifocal CIDP stays at CIDP. A similar distinction can be made between sensory CIDP and sensory‐predominant CIDP, even if the number of patients was quite small. A few case reports have described clinical deterioration after steroid therapy in sensory CIDP patients as well, and our findings are in agreement with this observation [31, 32]. On the other hand, sensory‐predominant CIDP patients displayed satisfactory treatment responses to steroids and a shorter disease duration at inclusion. This observation may be attributed to the evidence presented by long‐term follow‐up studies [13, 33], where subclinical motor NCS involvement was often observed as a transient stage preceding the manifestation of clinical weakness, leading to a shift in the clinical diagnosis of these patients. The finding of a low frequency of tremor in patients with sensory‐predominant CIDP is in contrast with a previous study that showed an increased risk of tremor in patients with inflammatory neuropathies who have better MRC scores. The etiology of tremor in immune‐mediated neuropathies, such as CIDP, remains elusive. Some authors propose that tremor originates centrally in the cerebellum [34]. Alternatively, there is supporting evidence for a peripheral origin of tremor. This is evidenced by observed correlations between tremor severity and nerve conduction parameters, including F‐wave latencies [34]. Further studies are needed to clarify the pathogenesis of tremor in CIDP and to confirm an increased risk in patients with some CIDP forms.

Limitations of our study include the retrospective nature of the data, possible selection bias, especially when dealing with nontypical cases, and lack of standardization in the conduction of NCS. Moreover, most of the patients included in the database were enrolled before the publication of the 2021 EAN/PNS criteria; therefore, response to treatment was confirmed using only an impairment or a disability measure. Finally, given the exploratory nature of this study and the relatively reduced sample size within CIDP variant subgroups, multiple testing correction was not applied.

This study confirms that the 2021 EAN/PNS criteria allow a clearer definition of the CIDP variants and the identification of some forms, multifocal and motor CIDP, with a different response to therapy compared to CIDP. It also confirms the reduced disability of patients with the CIDP variants compared to those with the typical form. This information, in clinical practice, allows for more precise individualization of therapy and more accurate counseling of patients regarding their prognosis. Moreover, a more precise definition of the individual variants could lead to easier diagnoses, preventing the insufficient recognition of these forms in clinical practice, which is one of the reasons for the frequent misdiagnosis of CIDP. Finally, the study confirms previous observations of a different distribution of electrophysiological abnormalities in peripheral nerves and a lower frequency of increased CSF proteins in patients with multifocal CIDP compared to typical CIDP, reinforcing the hypothesis of a less frequent proximal impairment in the multifocal compared to typical form.

## FUNDING INFORMATION

The study was supported by a grant from Regione Lombardia, Italy, for patients from this region and subsequently extended to other Italian centers and by a grant from Ministero della Salute, Ricerca Finalizzata (Progetto RF‐2016‐02361887). The study was also supported by unrestricted grants from Kedrion Biopharma (Italy), CSL Behring (Italy), Humanitas Clinical and Research Institute (Milan, Italy), and GBS‐CIDP Foundation International (USA). The funders had no role in study design, data collection and analysis, decision to publish, or the preparation of the manuscript.

## CONFLICT OF INTEREST STATEMENT

None of the authors has any conflict of interest to disclose.

## AUTHOR CONTRIBUTIONS

**Alberto De Lorenzo:** Conceptualization; writing – original draft; methodology; writing – review and editing; data curation; investigation; formal analysis. **Giuseppe Liberatore:** Conceptualization; methodology; data curation; writing – original draft; writing – review and editing; investigation. **Pietro Emiliano Doneddu:** Conceptualization; investigation; writing – original draft; methodology; writing – review and editing; data curation. **Fiore Manganelli:** Conceptualization; investigation; writing – review and editing. **Dario Cocito:** Conceptualization; investigation; writing – review and editing. **Chiara Briani:** Investigation; conceptualization; writing – review and editing. **Raffaella Fazio:** Conceptualization; investigation; writing – review and editing. **Anna Mazzeo:** Conceptualization; investigation; writing – review and editing. **Angelo Schenone:** Conceptualization; investigation; writing – review and editing. **Vincenzo Di Stefano:** Conceptualization; investigation; writing – review and editing. **giuseppe cosentino:** Conceptualization; investigation; writing – review and editing. **Girolama Alessandra Marfia:** Conceptualization; investigation; writing – review and editing. **L. Benedetti:** Conceptualization; investigation; writing – review and editing. **Marinella Carpo:** Investigation; conceptualization; writing – review and editing. **Massimiliano Filosto:** Conceptualization; investigation; writing – review and editing. **Giovanni Antonini:** Conceptualization; investigation; writing – review and editing. **Angelo Maurizio Clerici:** Conceptualization; investigation; writing – review and editing. **Marco Luigetti:** Conceptualization; investigation; writing – review and editing. **Sabrina Matà:** Conceptualization; investigation; writing – review and editing. **Tiziana Rosso:** Conceptualization; investigation; writing – review and editing. **Marta Lucchetta:** Conceptualization; investigation; writing – review and editing. **Gabriele Siciliano:** Conceptualization; investigation; writing – review and editing. **Giuseppe Lauria Pinter:** Conceptualization; investigation; writing – review and editing. **Guido Cavaletti:** Conceptualization; investigation; writing – review and editing. **Maurizio Inghilleri:** Conceptualization; investigation; writing – review and editing. **Teresa Cantisani:** Conceptualization; investigation; writing – review and editing. **Francesca Notturno:** Conceptualization; investigation; writing – review and editing. **Dario Ricciardi:** Conceptualization; investigation; writing – review and editing. **Francesco Habetswallner:** Conceptualization; investigation; writing – review and editing. **Emanuele Spina:** Conceptualization; investigation; writing – review and editing. **Erdita Peci:** Conceptualization; investigation; writing – review and editing. **Alessandro Salvalaggio:** Conceptualization; investigation; writing – review and editing. **Yuri Falzone:** Conceptualization; investigation; writing – review and editing. **Camilla Strano:** Conceptualization; investigation; writing – review and editing. **Luca Gentile:** Conceptualization; investigation; writing – review and editing. **Elisa Vegezzi:** Conceptualization; investigation; writing – review and editing. **Giorgia Mataluni:** Conceptualization; investigation; writing – review and editing. **Stefano Cotti Piccinelli:** Conceptualization; investigation; writing – review and editing. **Luca Leonardi:** Conceptualization; investigation; writing – review and editing. **Angela Romano:** Conceptualization; investigation; writing – review and editing. **Eduardo Nobile‐Orazio:** Conceptualization; investigation; writing – review and editing; data curation; methodology; writing – original draft; funding acquisition; supervision.

## Data Availability

The data that support the findings of this study are available from the corresponding author upon reasonable request.

## References

[1] Mathey EK , Park SB , Hughes RAC , et al. Chronic inflammatory demyelinating polyradiculoneuropathy: from pathology to phenotype. J Neurol Neurosurg Psychiatry. 2015;86(9):973‐985. doi:10.1136/jnnp-2014-309697 25677463 PMC4552934

[2] Viala K , Maisonobe T , Stojkovic T , et al. A current view of the diagnosis, clinical variants, response to treatment and prognosis of chronic inflammatory demyelinating polyradiculoneuropathy. J Peripher Nerv Syst. 2010;15(1):50‐56. doi:10.1111/j.1529-8027.2010.00251.x 20433605

[3] van den Berg‐Vos RM , van den Berg LH , Franssen H , et al. Multifocal inflammatory demyelinating neuropathy: a distinct clinical entity? Neurology. 2000;54(1):26‐32. doi:10.1212/wnl.54.1.26 10636121

[4] Viala K , Renié L , Maisonobe T , et al. Follow‐up study and response to treatment in 23 patients with Lewis‐Sumner syndrome. Brain. 2004;127(9):2010‐2017. doi:10.1093/brain/awh222 15289267

[5] Kuwabara S , Isose S , Mori M , et al. Different electrophysiological profiles and treatment response in “typical” and “atypical” chronic inflammatory demyelinating polyneuropathy. J Neurol Neurosurg Psychiatry. 2015;86(10):1054‐1059. doi:10.1136/jnnp-2014-308452 25424435

[6] Rajabally YA , Chavada G . Lewis‐Sumner syndrome of pure upper‐limb onset: diagnostic, prognostic, and therapeutic features. Muscle Nerve. 2009;39(2):206‐220. doi:10.1002/mus.21199 19145651

[7] Berger AR , Herskovitz S , Kaplan J . Late motor involvement in cases presenting as “chronic sensory demyelinating polyneuropathy”. Muscle Nerve. 1995;18(4):440‐444. doi:10.1002/MUS.880180411 7715630

[8] Sinnreich M , Klein CJ , Daube JR , Engelstad J , Spinner RJ , Dyck PJB . Chronic immune sensory polyradiculopathy: a possibly treatable sensory ataxia. Neurology. 2004;63(9):1662‐1669. doi:10.1212/01.WNL.0000142507.12763.58 15534252

[9] Katz JS , Saperstein DS , Gronseth G , Amato AA , Barohn RJ . Distal acquired demyelinating symmetric neuropathy. Neurology. 2000;54(3):615‐620. doi:10.1212/wnl.54.3.615 10680792

[10] Sabatelli M , Madia F , Mignogna T , Lippi G , Quaranta L , Tonali P . Pure motor chronic inflammatory demyelinating polyneuropathy. J Neural Transm. 2001;248(9):772‐777. doi:10.1007/s004150170093 11596782

[11] Oh SJ , Joy JL , Kuruoglu R . “Chronic sensory demyelinating neuropathy”: chronic inflammatory demyelinating polyneuropathy presenting as a pure sensory neuropathy. J Neurol Neurosurg Psychiatry. 1992;55(8):677‐680. doi:10.1136/jnnp.55.8.677 1326601 PMC489203

[12] Lewis RA , Sumner AJ , Brown MJ , Asbury AK . Multifocal demyelinating neuropathy with persistent conduction block. Neurology. 1982;32(9):958‐964. doi:10.1212/WNL.32.9.958 7202168

[13] Doneddu PE , Cocito D , Manganelli F , et al. Atypical CIDP: diagnostic criteria, progression and treatment response. Data from the Italian CIDP database. J Neurol Neurosurg Psychiatry. 2019;90(2):125‐132. doi:10.1136/JNNP-2018-318714 30297520

[14] Ikeda S , Koike H , Nishi R , et al. Clinicopathological characteristics of subtypes of chronic inflammatory demyelinating polyradiculoneuropathy. J Neurol Neurosurg Psychiatry. 2019;90(9):988‐996. doi:10.1136/JNNP-2019-320741 31227562

[15] Shimizu F , Oishi M , Sawai S , et al. Increased IP‐10 production by blood‐nerve barrier in multifocal acquired demyelinating sensory and motor neuropathy and multifocal motor neuropathy. J Neurol Neurosurg Psychiatry. 2019;90(4):444‐450. doi:10.1136/jnnp-2018-319270 30523038

[16] Beppu M , Sawai S , Misawa S , et al. Serum cytokine and chemokine profiles in patients with chronic inflammatory demyelinating polyneuropathy. J Neuroimmunol. 2015;279:7‐10. doi:10.1016/j.jneuroim.2014.12.017 25669993

[17] Pascual‐Goñi E , Fehmi J , Lleixà C , et al. Antibodies to the Caspr1/contactin‐1 complex in chronic inflammatory demyelinating polyradiculoneuropathy. Brain. 2021;144(4):1183‐1196. doi:10.1093/BRAIN/AWAB014 33880507

[18] Querol L . Autoimmune nodopathies: treatable neuropathies beyond traditional classifications. J Neurol Neurosurg Psychiatry. 2021;92(10):1025. doi:10.1136/JNNP-2021-326676 34400541

[19] Van den Bergh PYK , van Doorn PA , Hadden RDM , et al. European academy of neurology/peripheral nerve society guideline on diagnosis and treatment of chronic inflammatory demyelinating polyradiculoneuropathy: report of a joint task force‐second revision. Eur J Neurol. 2021;28(11):3556‐3583. doi:10.1111/ENE.14959 34327760

[20] Liberatore G , Manganelli F , Doneddu PE , et al. Chronic inflammatory demyelinating polyradiculoneuropathy: can a diagnosis be made in patients not fulfilling electrodiagnostic criteria? Eur J Neurol. 2020;28:620‐629. doi:10.1111/ene.14545 32959475

[21] Breiner A , Moher D , Brooks J , et al. Adult CSF total protein upper reference limits should be age‐partitioned and significantly higher than 0.45 g/L: a systematic review. J Neurol. 2019;266(3):616‐624. doi:10.1007/S00415-018-09174-Z 30617996

[22] Doneddu PE , Mandia D , Gentile F , et al. Home monitoring of maintenance intravenous immunoglobulin therapy in patients with chronic inflammatory neuropathy. J Peripher Nerv Syst. 2020;25(3):238‐246. doi:10.1111/JNS.12396 32470190

[23] Van Den Bergh PYK , Hadden RDM , Bouche P , et al. European Federation of Neurological Societies/peripheral nerve society guideline on management of chronic inflammatory demyelinating polyradiculoneuropathy: report of a joint task force of the European Federation of Neurological Societies and the peripheral nerve society—first revision. J Peripher Nerv Syst. 2010;15(1):1‐9. doi:10.1111/J.1529-8027.2010.00245.X 20433600

[24] Doneddu PE , Akyil H , Manganelli F , et al. Unclassified clinical presentations of chronic inflammatory demyelinating polyradiculoneuropathy. J Neurol Neurosurg Psychiatry. 2023;94:614‐621. doi:10.1136/JNNP-2022-331011 37015771

[25] Doneddu PE , De Lorenzo A , Manganelli F , et al. Comparison of the diagnostic accuracy of the 2021 EAN/PNS and 2010 EFNS/PNS diagnostic criteria for chronic inflammatory demyelinating polyradiculoneuropathy. J Neurol Neurosurg Psychiatry. 2022;93(12):1239‐1246. doi:10.1136/JNNP-2022-329357 36190959

[26] Rajabally YA , Afzal S , Loo LK , Goedee HS . Application of the 2021 EAN/PNS criteria for chronic inflammatory demyelinating polyneuropathy. J Neurol Neurosurg Psychiatry. 2022;93(12):1247‐1252. doi:10.1136/JNNP-2022-329633 36190956

[27] Shimizu F , Sawai S , Sano Y , et al. Severity and patterns of blood‐nerve barrier breakdown in patients with chronic inflammatory demyelinating polyradiculoneuropathy: correlations with clinical subtypes. PloS One. 2014;9(8):e104205. doi:10.1371/journal.pone.0104205 25105500 PMC4126720

[28] Larue S , Bombelli F , Viala K , et al. Non‐anti‐MAG DADS neuropathy as a variant of CIDP: clinical, electrophysiological, laboratory features and response to treatment in 10 cases. Eur J Neurol. 2011;18(6):899‐905. doi:10.1111/J.1468-1331.2010.03312.X 21199182

[29] Pegat A , Boisseau W , Maisonobe T , et al. Motor chronic inflammatory demyelinating polyneuropathy (CIDP) in 17 patients: clinical characteristics, electrophysiological study, and response to treatment. J Peripher Nerv Syst. 2020;25(2):162‐170. doi:10.1111/JNS.12380 32364302

[30] Van Schaik IN , Léger JM , Nobile‐Orazio E , et al. European Federation of Neurological Societies/peripheral nerve society guideline on management of multifocal motor neuropathy. Report of a joint task force of the European Federation of Neurological Societies and the peripheral nerve society—first revision. J Peripher Nerv Syst. 2010;15(4):295‐301. doi:10.1111/J.1529-8027.2010.00290.X 21199100

[31] Rajabally YA , Wong SL . Chronic inflammatory pure sensory polyradiculoneuropathy: a rare CIDP variant with unusual electrophysiology. J Clin Neuromuscul Dis. 2012;13(3):149‐152. doi:10.1097/CND.0b013e31822484fb 22538310

[32] Chroni E , Veltsista D , Gavanozi E , Vlachou T , Polychronopoulos P , Papathanasopoulos P . Pure sensory chronic inflammatory polyneuropathy: rapid deterioration after steroid treatment. BMC Neurol. 2015;15(1):1‐5. doi:10.1186/s12883-015-0291-7 25885891 PMC4359520

[33] Van Dijk GW , Notermans NC , Franssen H , Wokke JHJ . Development of weakness in patients with chronic inflammatory demyelinating polyneuropathy and only sensory symptoms at presentation: a long‐term follow‐up study. J Neurol. 1999;246(12):1134‐1139. doi:10.1007/s004150050531 10653304

[34] Saifee TA , Schwingenschuh P , Reilly MM , et al. Tremor in inflammatory neuropathies. J Neurol Neurosurg Psychiatry. 2013;84(11):1282‐1287. doi:10.1136/jnnp-2012-303013 22952325

